# Proteomic Analysis Reveals Proteins Involved in the Mode of Action of β-Citronellol Identified From *Citrus hystrix* DC. Leaf Against *Candida albicans*

**DOI:** 10.3389/fmicb.2022.894637

**Published:** 2022-05-23

**Authors:** Watunyoo Buakaew, Rungnapa Pankla Sranujit, Chanai Noysang, Sucheewin Krobthong, Yodying Yingchutrakul, Yordhathai Thongsri, Pachuen Potup, Krai Daowtak, Kanchana Usuwanthim

**Affiliations:** ^1^Cellular and Molecular Immunology Research Unit, Faculty of Allied Health Sciences, Naresuan University, Phitsanulok, Thailand; ^2^Faculty of Integrative Medicine, Rajamangala University of Technology Thanyaburi, Pathum Thani, Thailand; ^3^Interdisciplinary Graduate Program in Genetic Engineering, Kasetsart University, Bangkok, Thailand; ^4^Center for Neuroscience, Faculty of Science, Mahidol University, Bangkok, Thailand; ^5^National Omics Center, National Science and Technology Development Agency (NSTDA), Pathum Thani, Thailand

**Keywords:** β-citronellol, *Candida albicans*, antifungal, proteomics, biofilm formation

## Abstract

*Candida albicans* is a fungus that lives primarily on the mucosal surfaces of healthy humans, such as the oral cavity, vagina, and gastrointestinal tract. This commensal organism can be controlled by other microbiota, while certain conditions can increase the risk of *C. albicans* outgrowth and cause disease. Prevalence of the drug-resistant phenotype, as well as the severity of *C. albicans* infection in immunocompromised patients, presents a challenge for scientists to develop novel, effective treatment, and prevention strategies. β-Citronellol is an intriguing active compound of several plants that has been linked to antifungal activity, but data on the mechanism of action in terms of proteomic profiling are lacking. Here, β-citronellol identified from *Citrus hystrix* DC. leaf against *C. albicans* were evaluated. A proteomic approach was used to identify potential target proteins involved in the mode of action of β-citronellol. This study identified and discussed three protein groups based on the 126 major proteins that were altered in response to β-citronellol treatment, 46 of which were downregulated and 80 of which were upregulated. Significant protein groups include cell wall proteins (e.g., Als2p, Rbt1p, and Pga4p), cellular stress response enzymes (e.g., Sod1p, Gst2p, and Ddr48p), and ATP synthesis-associated proteins (e.g., Atp3p, Atp7p, Cox1p, and Cobp). Results demonstrated the complexities of protein interactions influenced by β-citronellol treatment and highlighted the potential of antifungal activity for future clinical and drug development research.

## Introduction

*Candida* species form part of the human microbiota and colonize various parts of the body such as the skin, gastrointestinal tract, oral cavity and reproductive tract (Cannon and Chaffin, 1999; Achkar and Fries, 2010; Kumamoto, 2011; Kühbacher et al., 2017). There are several *Candida* species including *Candida albicans*, *Candida glabrata*, *Candida parapsilosis*, *Candida tropicalis*, *Candida famata*, and *Candida krusei* (de Oliveira Santos et al., 2018). The most dominant and frequently isolated species from human samples is *C. albicans* (Pfaller and Diekema, 2007; Wächtler et al., 2012; Quindós, 2014). In healthy individuals, growth of *C. albicans* is controlled by other members of the human microbiota. Some factors such as host-microbiome imbalance, changes in host immune response and variation in the local environment can increase the risk of *C. albicans* infection (Nobile and Johnson, 2015). *Candida* species infection is a serious threat to public health around the world, particularly in immunocompromised people (Low and Rotstein, 2011; Eades and Armstrong-James, 2019). This opportunistic fungus can cause a variety of diseases in susceptible patients depending on where it colonizes the body including oral candidiasis (Wächtler et al., 2012) and vaginal candidiasis (Peters et al., 2014). Additionally, systemic infection with *Candida* species is associated with a high mortality rate of 46–75% in cases of invasive candidiasis (Pfaller and Diekema, 2007). Good management and therapeutic approaches to *Candida* infection control are required to reduce the severity of infection.

Several antifungal agents are commonly used to combat *Candida* infections. Antifungal drugs are classified into several groups including azoles, polyenes, allylamines, echinocandins, nucleoside analogs, and mitotic inhibitors (Lewis, 2011). With high drug efficacy and availability in a variety of formulations, azoles are commonly used for various *Candida* infections (Pappas et al., 2004; de Oliveira Santos et al., 2018). Several previous studies found an increase in antifungal drug resistance mechanisms in *Candida* species, including the azoles group (Cowen et al., 2014; Morace et al., 2014; Bhattacharya et al., 2020). Therefore, finding alternative antifungal substances could be a promising approach for treatment of fungal infections. Several studies have demonstrated the potential therapeutic effect of plant compounds for anti-*Candida* activity (Prabhakar et al., 2008; Soliman et al., 2017; Suurbaar et al., 2017; Bonifácio et al., 2019). β-citronellol is an acyclic monoterpene that occurs naturally in volatile oil from certain types of plants including *Citrus hystrix* DC. (*C. hystrix* DC.), *Cymbopogon citratus* (DC) Stapf. (Poaceae), and *Vitis vinifera* L (Vasconcelos et al., 2016). Due to the pleasant odor and flavor, this active compound has been used in the perfume and food industries (Nerio et al., 2010; Vasconcelos et al., 2016). Our previous work identified citronellol from *n*-hexane and ethanolic crude extracts of the leaf part of *C. hystrix*, which demonstrated 1.42 and 4.28% relative mass, respectively (Ho et al., 2020; Buakaew et al., 2021a,b). The relative mass of an interesting citronellol was defined as its peak area divided by the total area of peaks in a single running sample. The concentration of citronellol in these crude extracts of *C. hystrix* DC. leaf was shown by the peak region. The bioactivity of β-citronellol has been reported as having repellant (Michaelakis et al., 2014), antibacterial (Nostro et al., 2013), anti-inflammation (Brito et al., 2012), anticancer (Ho et al., 2020), anti-allergic (Kobayashi et al., 2016), and antifungal properties (Pereira et al., 2015; Sharma et al., 2020a). A few reports have investigated the potential effect of β-citronellol on *C. albicans* (Silva et al., 2020; Sharma et al., 2020b). However, information on the molecular mechanism underlying global protein expression remains lacking. This study investigated the changes in protein expression using a proteomics approach, as a useful method to determine the whole protein alteration of *C. albicans* in response to β-citronellol treatment. Findings revealed that cell wall proteins, e.g., cell wall protein RTB1 (Rbt1p), agglutinin-like protein 2 (Als2p), and 1,3-beta-glucanosyltransferase PGA4 (Pga4p), cellular oxidative stress response, e.g., superoxide dismutase [Cu-Zn] (Sod1p), and glutathione S-transferase (Gst2p), and stress protein DDR48 (Ddr48p), and ATP synthesis proteins, e.g., ATP synthase subunit gamma (Atp3p), ATP synthase subunit d, mitochondrial (Atp7p), cytochrome *c* oxidase subunit 1 (Cox1p), and cytochrome *b* (Cobp) were the key targets influenced by β-citronellol as well as other protein groups which might be involved in the mode of action.

## Materials and Methods

### Plant Material Preparation and Extraction of *Citrus hystrix* Leaves

*Citrus hystrix* DC leaves powder obtained from Khaolaor Company (Samut Prakan, Thailand) was macerated in three different solvents sequentially, as previously described (Buakaew et al., 2021a,b). Crude hexane, crude ethyl acetate, and crude ethanol extracts were obtained. Citronellol, the active compound was identified from *C. hystrix* leaves extract (Buakaew et al., 2021a,b). For *in vitro* experiments, citronellol was purchased from Sigma-Aldrich Company (St. Louis, MO, United States). They were stored at 4°C for future experiments.

### Culture and Growth of *Candida albicans*

*C. albicans* ATCC 90028 was used as the model. Yeast inoculum was prepared from 24 h colonies on Sabouraud dextrose agar (SDA: HiMedia Laboratories, Mumbai, India). The cell suspension was adjusted in different broth media using a spectrophotometer (A_600_nm) at a final concentration of 10^6^ cells/ml for cytotoxicity and biofilm formation assays. For proteomic analysis and other methods, yeast cells were adjusted to 10^7^ cells/ml for final concentration.

### Calculation of Minimal Inhibitory Concentration and Minimum Fungicidal Concentration

A broth microdilution test was carried out to determine the minimal inhibitory concentration (MIC) value of all crude extracts and β-citronellol according to the Clinical and Laboratory Standards Institute (CLSI) guidelines M27-A2 with some modifications. In brief, 100 μl of 2-fold serial dilution of crude extracts (0.20–100 mg/ml) and β-citronellol (2–1,024 μg/ml) in RPMI-1640 medium (Thermo Fisher Scientific, Waltham, MA, United States) were added to a 96-well plate. The suspension of *C. albicans* was adjusted at 530 nm to match the turbidity of 0.5 McFarland standard. Then, 10 μl of yeast cell suspension was added to each well of pre-diluted crude extracts and β-citronellol to reach the final concentration of 0.5–1 × 10^3^ cells/ml. The cells were then incubated at 35 ± 2°C for 48 h. After incubation, 100 μl of resazurin (0.02 mg/ml) was added and further incubated for 3 h at 35 ± 2°C. A change in color was observed and wells with no change in color (the blue color of resazurin remained unchanged) were evaluated as MIC values. While the spread plate technique was used to establish the minimum fungicidal concentration (MFC). Ten microliter of cell suspension was spread on SDA and incubated at 35 ± 2°C for 48 h. The MFC was determined based on the concentration in the absence of colony growth.

### Kinetic Growth Inhibition Assay

To determine the effect of β-citronellol on yeast growth, the cells were incubated in 100 μl of Sabouraud dextrose broth (SDB: HiMedia Laboratories, Mumbai, India) containing different concentrations of β-citronellol in a 96-well plate. The plate was incubated at 35 ± 2°C for 48 h in a multidetector microplate reader. The turbidity was measured at OD_600 _nm every 2 h.

### Sample Preparation for Proteomic Analysis

To prepare *C. albicans* treated with β-citronellol for proteomic analysis, the yeast cells were incubated with β-citronellol (128 μg/ml) in SDB for 24 h. The treated cells were washed twice with 1× phosphate-buffered saline (PBS) pH 7.4 (137 mM NaCl, 2.7 mM KCl, 10 mM Na_2_HPO_4_, and 1.8 mM KH_2_PO_4_). After that, the cells were resuspended with lysis buffer [1% SDS, 5 mM dithiothreitol (DTT) in 0.1× PBS pH 7.4 (13.7 mM NaCl, 0.27 mM KCl, 0.43 mM Na_2_HPO_4_, and 0.147 mM KH_2_PO_4_) with 1× protease inhibitor cocktail], sonicated for 30 s and rested on ice for 30 s × 5 rounds. Then, the cells were centrifuged at 12,000 RPM, 4°C for 20 min. Proteins extracted were precipitated using ice-cold acetone and stored at −20°C for 16 h. After precipitation, the protein pellet was reconstituted in 0.25% RapidGest SF (Waters, United Kingdom) in 10 mM ammonium bicarbonate. The protein concentrations were determined by the bicinchoninic acid assay kit (Pierce, New York, NY, United States), using bovine serum albumin as the standard. Then, 25 μg of protein were subjected to gel-free based digestion. Reduction of sulfhydryl bonds was performed using 5 mM DTT, with incubation at 80°C for 20 min. Alkylation of sulfhydryl groups was performed using 25 mM iodoacetamide (IAA) at room temperature for 30 min in the dark. The solution was cleaned-up using a 7 kDa molecular weight cut-off, 0.5 ml (Zeba™ Spin Desalting Columns, Thermo Fisher, United States). Proteolytic digestion using 500 ng of sequencing grade trypsin (Promega, Germany) was added to the protein solution and incubated at 37°C for 3 h. The peptides were resolubilized in 0.1% formic acid/LC–MS water before transfer to a TruView LC-MS vial (Waters, United Kingdom).

### Proteomic Analysis by Liquid Chromatography-Tandem Mass Spectrometry (LC-MS/MS)

To investigate the protein expression profiles of *C. albicans*, the peptide mixtures were analyzed in data-dependent mode on tandem mass spectrometers using an Orbitrap HF hybrid mass spectrometer combined with an UltiMate 3000 LC system. Briefly, the peptides were first desalted on line on a reverse-phase C18 PepMap 100 trapping column, before being resolved onto a C18 PepMapTM 100 capillary column with a 150-min gradient of CH_3_CN, 0.1% formic acid at a flow rate of 300 nl/min. Peptides were analyzed by applying a data-dependent Top15 method consisting of a scan cycle initiated by a full scan of peptide ions in the Orbitrap analyzer, followed by high-energy collisional dissociation and MS/MS scans of the 15 most abundant precursor ions. Full scan mass spectra were acquired from *m/*z 400–1,600 with an AGC target set at 3 × 10^6^ ions and resolution of 60,000. The MS/MS scan was initiated when the ACG target reached 10^5^ ions. Ion selection was performed by applying a dynamic exclusion window of 12 s.

Raw files were analyzed by Proteome Discoverer software version 2.4 (Thermo Scientific) using the SEQUEST, Percolator and Minora algorithms. The LC-MS spectrum was matched against the UniProt C. *albicans* reference proteome (UP000000559; downloaded 08/08/2021; 6,035 entries). For protein identification and quantification, the setting parameters were as follows: a maximum of two trypsin missed cleavages were allowed with a precursor mass tolerance of 5 ppm and fragment mass tolerance of 0.01 Da. Carbamidomethylating +57.021 Da (cysteine) and oxidation +15.995 Da (methionine) were chosen as static modifications and oxidation +15.995 Da (methionine) were chosen as dynamic modifications. The false discovery rate (FDR) for peptide and protein identification was set to 0.05 in both cases. Normalization of the relative protein abundance ratio was performed by the total peptide amount for each LC-run (across all runs; *n* = 6) by the normalization algorithm of Proteome Discoverer software. To assemble the differentially expressed protein list, multiple consensus workflows were used within the Proteome Discoverer software to assemble the PSMs into peptide groups, protein database matches and finally non-redundant protein groups using the principle of strict parsimony as defined by the vendor software defaults. The mass spectrometry proteomics data have been deposited to the ProteomeXchange Consortium *via* the PRIDE (Perez-Riverol et al., 2019) partner repository with the dataset identifier PXD030379 and 10.6019/PXD030379.

### Biofilm Formation Assay and Crystal Violet Staining

To determine the effect of β-citronellol on biofilm formation, *C. albicans* biofilm formation assay was performed according to the protocol previously described (Gulati et al., 2018). Briefly, 48 h of *C. albicans* colonies on SDA were adjusted in RPMI-1640 to obtain 10^6^ CFU/ml. Then, 100 μl of yeast was added to a flat-bottom cell culture 96-well plate (Wuxi NEST Biotechnology Co., Ltd., Jiangsu, China) and incubated at 35 ± 2°C for 1.5 h with constant shaking at 250 RPM. The plate was washed once with PBS to remove non-adherent cells. To determine the effects of active compounds, 100 μl of different concentrations of citronellol were added and incubated at 35 ± 2°C for 24 h. The cells treated with 2 μg/ml of amphotericin B were served as positive control. After incubation, biofilm from *C. albicans* was rinsed three times with PBS to eliminate non-adherent cells. The plate was air-dried for 45 min at room temperature. Then, yeast biofilm was stained with 0.4% crystal violet for 45 min and washed three times with PBS. To measure the absorbance, 200 μl of 95% ethanol was added to destain crystal violet for 45 min and 100 μl of each well was transferred to a new plate. The absorbance was measured at OD_595 _nm using a microplate reader.

### Determination of Reactive Oxygen Species Production

To determine the production of reactive oxygen species (ROS), cell were treated with β-citronellol and the level of ROS was measured using 2′,7′-dichlorofluorescein diacetate (H_2_DCFDA; Invitrogen, Waltham, MA, United States) following the manufacturer’s protocol. *C. albicans* (10^6^ cells/ml) was incubated at 35 ± 2°C for 4 h with different concentrations of β-citronellol in SDB. Yeast cells treated with 2 mM hydrogen peroxide (H_2_O_2_) in SDB was used as positive control in this experiment. After incubation, cells were collected by centrifuging at 12,000 RPM for 5 min and washed once with PBS. Cells were incubated with 10 μM H_2_DCFDA in PBS at 35 ± 2°C for 30 min in the dark. Then, cells were washed once with PBS and resuspended in the same buffer. One hundred microliter of cell suspensions were transferred to a 96-well fluorescence plate. The fluorescence intensity was measured at excitation/emission at 485/535 nm.

### Evaluation of Membrane Potential Disruption

To determine the effect of β-citronellol on yeast membrane potential, DiBAC_4_(3) [Bis-(1,3-dibutylbarbituric acid) trimethine oxonol; Invitrogen, Waltham, MA, United States] was used to determine the cell membrane potential as described by Yang et al. (2019). Briefly, yeast cells (10^6^ cells/ml) were prepared in SDB, then incubated with diluted β-citronellol at 35 ± 2°C for 4 h. For positive control group, yeast cells were treated with 2 μg/ml of amphotericin B. The cells were harvested by centrifuging at 12,000 RPM for 5 min and washed once with PBS. After that, yeast cells were incubated at 35 ± 2°C in the dark with 20 μg/ml DiBAC_4_(3) in PBS. After incubation, the cells were centrifuged and resuspended in PBS and transferred to a 96-well fluorescence plate. Fluorescence intensities were measured with a fluorescence spectrophotometer at excitation/emission wavelengths at 492/518 nm.

### Apoptosis Analysis in Yeast Cells

To determine the effect of β-citronellol on inducing programmed cell death by apoptosis, protoplast preparation was conducted following a previously described protocol (Lone et al., 2020) and protoplast buffer preparation (Khan et al., 2014). Briefly, cells exposed with various concentrations of β-citronellol (64, 128, and 256 μg/ml) for 6 h were washed twice with protoplast buffer-I (1 M sorbitol, 30 mM DTT, 50 mM Tris base, and 10 mM MgCl_2_), then incubated in the same buffer at 25°C for 15 min, followed by centrifugation at 12,000 RPM for 5 min. The pellets were resuspended with protoplast buffer-II (1 M sorbitol, 1 mM DTT, 50 mM Tris base, and 10 mM MgCl_2_) supplemented with lyticase enzyme (≥1 μg/ml; Sigma-Aldrich, MO, United States) and incubated at 25°C for 1 h. Then, the cells were centrifuged to remove buffer-II, followed by incubation in protoplast buffer-III (1 M sorbitol, 50 mM Tris base, and 10 mM MgCl_2_) at 25°C for 20 min. After incubation, cells were centrifuged, and supernatants were discarded and washed once with PBS. To examine the apoptosis phenotype, the Muse™ Annexin V & Dead Cell Kit (Merck, Darmstadt, Germany) was used. The protoplasts were resuspended in SDB with 1% fetal bovine serum (FBS) and 100 μl of protoplasts were mixed with 100 μl of the Muse™ Annexin V & Dead Cell Kit. The mixture was incubated at room temperature for 20 min in the dark. After that, yeast cells were analyzed using Muse™ Cell Analyzer following the manufacturer’s protocol.

### Scanning Electron Microscopy

To study the effect of β-citronellol on *C. albicans* morphology, tested yeast cells was observed using Scanning Electron Microscopy (SEM) according to a previously described protocol (Fischer et al., 2012). The cells were incubated with various concentrations of β-citronellol at 35 ± 2°C for 4 h, and then the suspension was centrifuged at 12,000 RPM for 5 min. The pellets were resuspended in PBS and 50 μl of cells were dropped on a glass slide and incubated at room temperature for 30 min. After that, 0.5 ml of primary fixative (4% paraformaldehyde) was added, followed by incubation at room temperature for 1 h. The cells were washed three times (2 min each), then post-fixed with 1% OsO_4_ at room temperature for 1 h. The post-fixed cells were washed once with PBS and dehydrated using 25, 50, 75, 95, and 100% ethanol. Then, the cells were dehydrated using 1:1 hexamethyldisilazane (HMDS): ethanol for 15 min, followed by 100% HMDS for 15 min. The excess volume of solution was wiped away, and the cells were dried at room temperature for 4 h. Then, the cells were gold-covered by cathodic spraying, and the morphology of yeast cells was observed 5,000× under Apreo 2 SEM (Thermo Scientific) in high vacuum mode at 5 kV.

### Statistical Analysis

Statistical analyses of all data were performed using GraphPad Prism version 8.01 (GraphPad Software, San Diego, CA, United States). A one-way analysis of variance (ANOVA) was used to determine the variables significance, with comparison of means by Tukey’s range test. All tests were performed in triplicate, and data were expressed as mean ± standard error. The *p*-value ≤0.05, was considered statistically significant. ^*^p ≤ 0.05, ^**^p ≤ 0.01, ^***^p ≤ 0.001, and ^****^p ≤ 0.0001.

## Results

### The Antifungal Activity of Crude Extracts and Active Compound on *Candida albicans*

The purpose of this experiment was to determine the MIC and MFC values for all crude *C. hystrix* DC. leaf extracts prepared using our extraction protocol and to investigate the relationship between these values and the amount of β-citronellol in each extract. The yeast cells were treated with different concentrations of crude extracts (0.2–100 mg/ml) and β-citronellol (2–1,024 μg/ml) at 35 ± 2°C for 48 h. In crude extracts group, the extract from ethanol had the lowest MIC value of 50 mg/ml, while the MIC values from *n*-hexane and ethyl acetate were equal to or greater than 100 mg/ml. However, the MFC of all three crude extracts was greater than 100 mg/ml. This result was related to the higher relative mass percentage of β-citronellol in ethanolic extract than *n*-hexane found in our previous studies (Ho et al., 2020; Buakaew et al., 2021a,b). The MIC and MFC values for β-citronellol treatment were 128 and 256 g/ml, respectively, as shown in Table 1. Based on these findings, β-citronellol was chosen as the candidate compound for further investigation.

**Table 1:** The minimum inhibitory concentration (MIC) and minimum fungicidal concentration (MFC) values against *Candida albicans*.

Treatment compound	MIC	MFC
Crude extract (*n*-hexane)	>100 mg/ml	>100 mg/ml
Crude extract (ethyl acetate)	>100 mg/ml	>100 mg/ml
Crude extract (ethanol)	50 mg/ml	>100 mg/ml
β-citronellol	128 μg/ml	256 μg/ml

### Growth Inhibition Kinetic Assay

**Figure 1:**
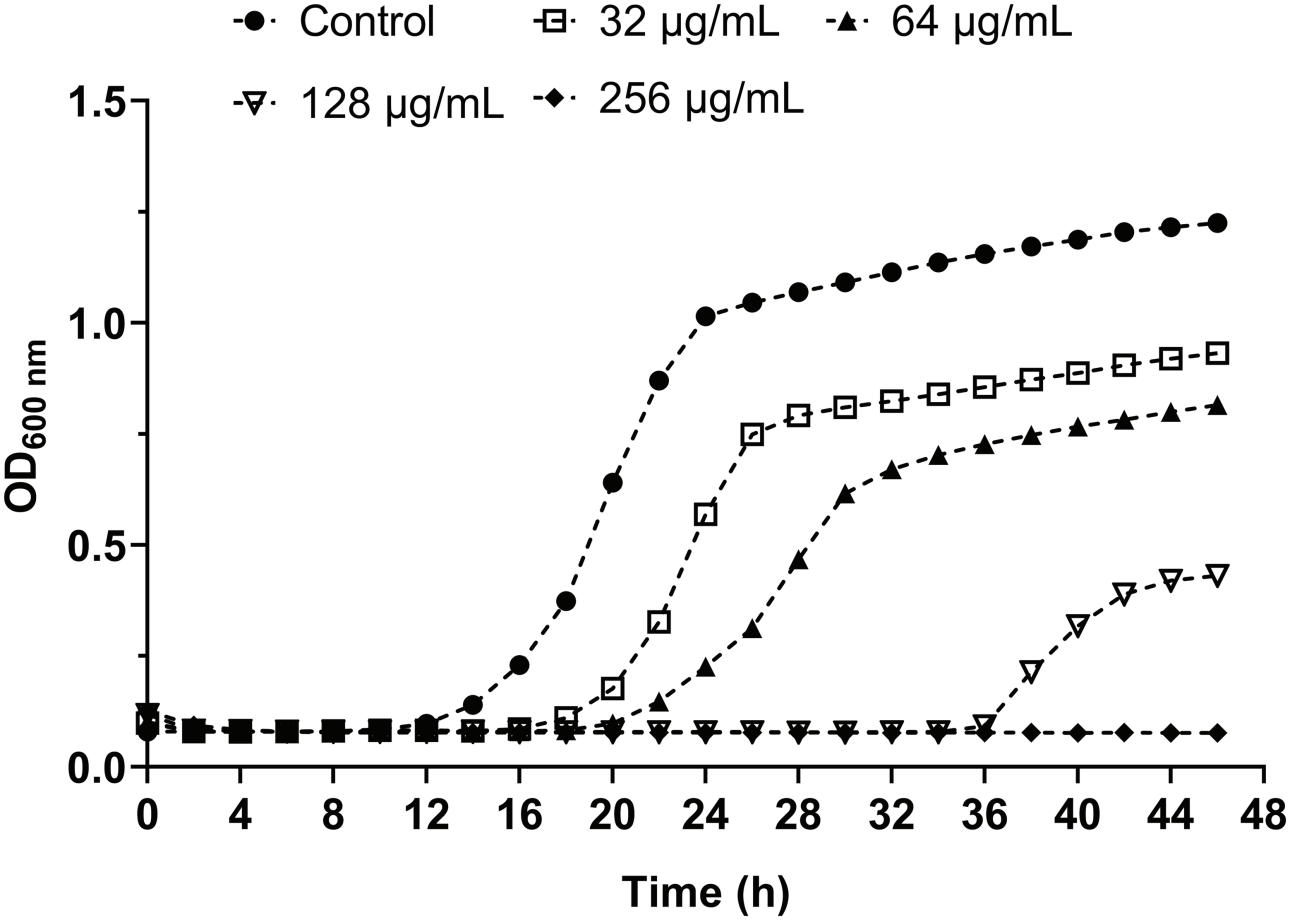
Effect of β-citronellol on *Candida albicans* kinetic growth curve. *Candida albicans* (1 × 10^3^ cells/ml) was treated with and without different concentrations of β-citronellol (32–256 μg/ml) at 35 ± 2°C for 48 h. Cell culture in each condition was measured for absorbance at 600 nm every 2 h. All data are expressed as the mean of three replicates.

To evaluate the influence of β-citronellol on the growth curve of yeast cells, the growth inhibitory effect of various concentrations of β-citronellol (32–256 μg/ml) on yeast cells is shown in Figure 1. Compared to the control, the β-citronellol treated condition showed significantly delayed cell growth with decreasing absorbance at 600 nm in a dose-dependent manner. The MIC and MFC values of β-citronellol on *C. albicans* ATCC 90028 were 128 and 256 μg/ml, respectively.

### Effect of β-Citronellol on *Candida albicans* Cellular Proteome

**Figure 2:**
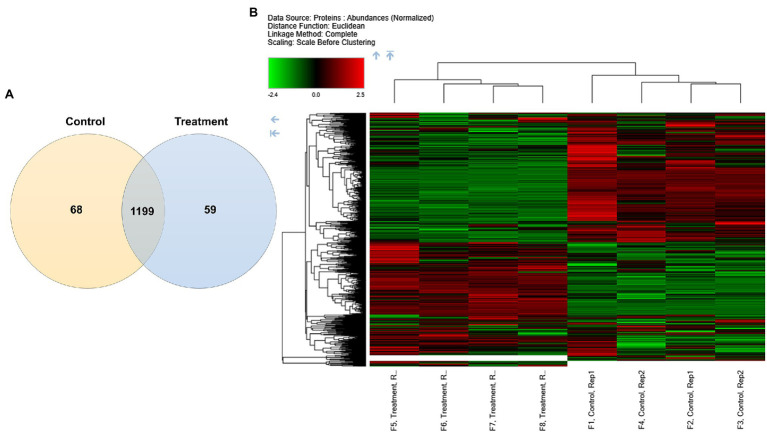
Differential protein expression profiles in *Candida albicans.* **(A)** Venn diagram depicting numbers of unique and common proteins identified in the control and treatment groups. **(B)** Heatmap with hierarchical clustering of differentially expressed proteins. Color key expression: green and red represent lower and higher differential abundance, respectively.

To investigate the protein expression profiles of *C. albicans*, yeast cells were treated with 128 μg/ml of β-citronellol for 24 h. Proteomics data were obtained using LC-MS/MS, while post-sample analysis was performed to generate high-certainty and high-specificity protein lists. Mass deviation showed that 94.93% of identified peptide groups had ≤10 ppm. For digestion specificity, 91.44% of all identified peptides had no miscleavage site, while 8.35% had a miscleavage site. In this study, the false discovery rate was set to <0.05. Overall, the total number of identified proteins was 1,326 from the control and treatment groups. As shown in Figure 2A, proteins commonly found among the two groups numbered 1,199 and unique proteins identified in the treatment and control groups numbered 59 and 68, respectively. Different expression proteins in each group were clustered hierarchically, as represented in the heatmap (Figure 2B). The columns show relative protein expression, while rows present significantly expressed proteins with maximum distance = 1.0.

**Figure 3:**
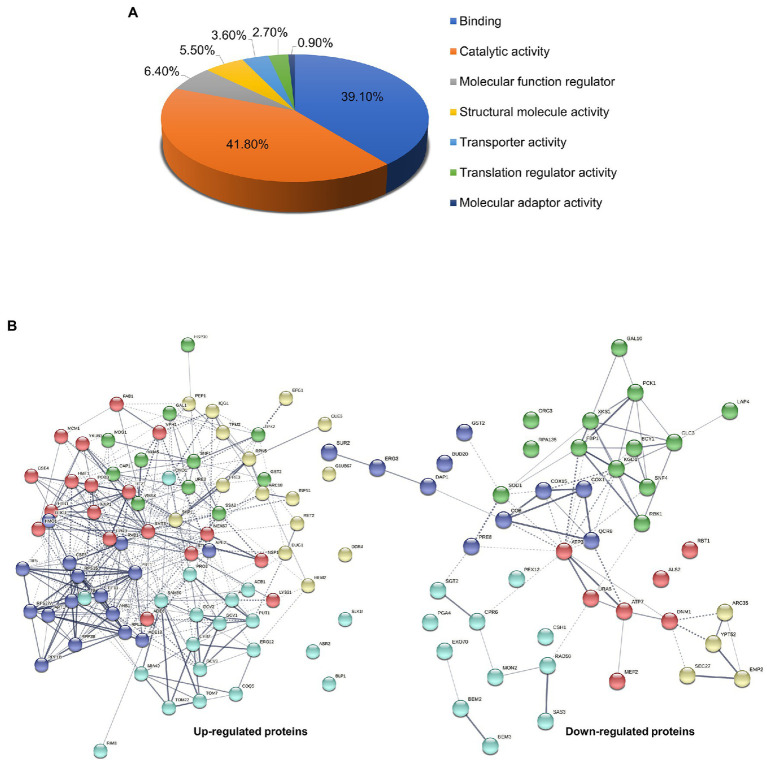
Gene ontology classification and protein—protein interaction network analysis of *Candida albicans* influenced by β-citronellol treatment. **(A)** Molecular function group classification of both up- and downregulated proteins by PANTHER-GO database. **(B)** Prediction maps of protein-protein interaction network by STRING v.11 analysis. The interaction network nodes represents down- and upregulated protein found in this study, which illustrated the potential molecular pathways involved in the mode of action of β-citronellol. The colors of each node represent protein clusters as determined by the kmeans clustering algorithm.

Based on statistical significance (*p* < 0.05) with differential expression ≥1.5-fold from the treatment group compared to the control, 126 significant altered proteins were functionally classified using PANTHER-GO Slim Molecular Function analysis tool. Results showed that altered proteins were mainly involved in catalytic activity (41.8%), binding (39.1%), molecular function regulator (6.4%), structural molecule activity (5.5%), transporter activity (3.6%), translation regulator activity (2.7%), and molecular adaptor activity (0.9%; Figure 3A). Protein-protein interactions in both up-regulated and down-regulated proteins were analyzed using the STRING v.11 database with a confidence score of 0.4 (Figure 3B and Table 2). Several pathways involved in the mode of action on killing *C. albicans* based on the 126 significant proteins altered in response to β-citronellol treatment. Among 46 downregulated and 80 upregulated proteins, the yeast cells exposed to β-citronellol suppress 10 interesting proteins, as shown in Table 3. These proteins include cell wall-associated proteins, such as cell wall protein RTB1 (Rbt1p; encoded by the gene *RBT1*), agglutinin-like protein 2 (Als2p; encoded by the gene *ALS2*), and 1,3-beta-glucanosyltransferase PGA4 (Pga4p; encoded by the gene *PGA4*). In addition, the treatment downregulates proteins involved in cellular response to oxidative stress, such as superoxide dismutase [Cu-Zn] (Sod1p; encoded by the gene *SOD1*), glutathione S-transferase (Gst2p; encoded by the gene *GST2*), and stress protein DDR48 (Ddr48p; encoded by the gene *DDR48*). Furthermore, the expression of ATP synthesis related proteins was decreased, such as ATP synthase subunit gamma (Atp3p; encoded by the gene *ATP3*), ATP synthase subunit d, mitochondrial (Atp7p; encoded by the gene *ATP7*), cytochrome *c* oxidase subunit 1 (Cox1p; encoded by the gene *COX1*), and cytochrome *b* (Cobp; encoded by the gene *COB*). In contrast, β-citronellol treatment upregulated the expression of proteins involved in various pathways, including heat shock proteins, such as heat shock protein SSA2 (Ssa2p), Hsp30p, and Hsp90 cochaperone (Sti1p), and ribosomal proteins, such as 40S ribosomal protein S27 (Rps27Ap), ribosomal protein P1B (Rpp1Bp), 60S ribosomal protein L13 (Rpl13p), ribosomal protein L37 (Rpl37Bp), and ribosomal 40S subunit protein S15 (Rps15p).

**Table 2:** List of protein expression in *Candida albicans* in β-citronellol treatment condition identified using LC-MS/MS.

Protein name	Gene name	Log_2_ fold change	Adjusted *p*-value
MRX complex DNA-binding subunit	*RAD50*	−18.16	3.01217E-15
Bud20p	*BUD20*	−17.69	1.01427E-14
Mon2p	*MON2*	−17.20	5.85441E-13
AMP-activated serine/threonine-protein kinase regulatory subunit	*SNF4*	−17.10	2.35381E-16
Cell wall protein RTB1	*RBT1*	−16.69	2.74374E-16
Agglutinin-like protein 2	*ALS2*	−15.77	5.57978E-13
Rab family GTPase	*YPT52*	−15.42	1.28246E-15
Bem2p	*BEM2*	−15.32	2.14077E-14
Exocyst complex protein EXO70	*EXO70*	−14.32	1.01427E-14
Origin recognition complex subunit 3	*ORC3*	−6.21	1.81551E-10
Histone acetyltransferase	*SAS3*	−4.56	7.62449E-06
Ribosome-releasing factor 2, mitochondrial	*MEF2*	−4.20	0.006295396
Peroxin-12	*PEX12*	−3.79	0.000115408
Coatomer subunit beta’	*SEC27*	−2.89	2.76378E-06
Proteasome subunit beta type-7	*PRE4*	−2.73	7.39922E-08
Sgt2p	*SGT2*	−2.32	2.02162E-07
Uridylate kinase	*URA6*	−2.10	8.06507E-09
Lap4p	*LAP4*	−1.78	5.5872E-05
Gst2p	*GST2*	−1.37	1.68003E-06
Superoxide dismutase [Cu-Zn]	*SOD1*	−1.28	0.037257003
ATP synthase subunit d, mitochondrial	*ATP7*	−1.23	4.45613E-05
Cox15p	*COX15*	−1.14	2.09369E-06
DNA-directed RNA polymerase subunit beta	*RPA135*	−1.07	3.75991E-06
Ribokinase	*RBK1*	−1.02	3.82379E-07
Cytochrome *b*	*COB*	−0.91	0.000534221
1,3-beta-glucanosyltransferase PGA4	*PGA4*	−0.89	1.78797E-05
Oxoglutarate dehydrogenase (succinyl-transferring)	*KGD1*	−0.88	5.89219E-07
Aldo-ket-red domain-containing protein	*CSH1*	−0.85	2.65849E-06
Fructose-bisphosphatase	*FBP1*	−0.83	7.59687E-06
Arp2/3 complex 34 kDa subunit	*ARC35*	−0.82	0.000664191
Phosphoenolpyruvate carboxykinase (ATP)	*PCK1*	−0.82	4.55772E-05
Bifunctional UDP-glucose 4-epimerase/aldose 1-epimerase	*GAL10*	−0.81	4.25493E-06
Cytochrome *c* oxidase subunit 1	*COX1*	−0.79	2.03346E-06
Proteasome core particle subunit alpha 2	*PRE8*	−0.76	1.85623E-05
Bem3p	*BEM3*	−0.76	0.000106366
Dap1p	*DAP1*	−0.76	0.007644593
Sphingosine hydroxylase	*SUR2*	−0.74	5.90462E-05
ATP synthase subunit gamma	*ATP3*	−0.72	3.75468E-06
Ubiquinol-cytochrome-*c* reductase subunit 8	*QCR8*	−0.70	0.003025807
Delta(7)-sterol 5(6)-desaturase ERG3	*ERG3*	−0.68	0.00074813
Emp24p	*EMP24*	−0.65	2.97306E-05
Xylulokinase	*XKS1*	−0.64	0.000211347
cAMP-dependent protein kinase regulatory subunit	*BCY1*	−0.64	1.16396E-05
Peptidyl-prolyl cis-trans isomerase D	*CPR6*	−0.63	9.96752E-06
Dynamin-related GTPase	*DNM1*	−0.60	4.19258E-05
1,4-alpha-glucan-branching enzyme	*GLC3*	−0.60	0.00016763
Phosphoinositide 5-phosphatase	*INP51*	18.96	3.80205E-17
Enhanced filamentous growth protein 1	*EFG1*	17.01	4.09147E-13
Protein-arginine omega-N methyltransferase	*HMT1*	16.53	1.28902E-07
mRNA export factor MEX67	*MEX67*	16.20	1.64181E-15
AP-1-like transcription factor CAP1	*CAP1*	15.62	1.48422E-14
Golgi to ER traffic protein 2	*GET2*	15.30	1.28246E-15
Delta-aminolevulinic acid dehydratase	*HEM2*	14.85	1.28246E-15
FG-nucleoporin	*NSP1*	14.49	4.88599E-16
Tom7p	*TOM7*	4.11	2.51163E-08
V-type proton ATPase subunit D	*VMA8*	3.65	9.90528E-08
Long-chain fatty acid transporter	*ACB1*	3.00	8.15303E-09
Eukaryotic translation initiation factor 4C	*TIF11*	2.94	2.5387E-09
Calmodulin	*CMD1*	2.89	8.15303E-09
Actin-related protein 2/3 complex subunit 3	*ARC18*	2.64	2.63026E-10
Tropomyosin	*TPM2*	2.56	2.0898E-06
GTP-binding protein	*DRG1*	2.51	6.15809E-07
Tom22p	*TOM22*	2.27	3.76269E-08
Blood-induced peptide 1	*BLP1*	2.17	2.51013E-08
Stress protein DDR48	*DDR48*	2.15	4.11631E-06
Mevalonate kinase	*ERG12*	1.81	7.99628E-08
Glycine cleavage system P protein	*GCV2*	1.77	8.81781E-06
Ubiquitin-binding protein	*CUE5*	1.77	6.72245E-07
Proline dehydrogenase	*PUT1*	1.62	7.40773E-06
Mdg1p	*MDG1*	1.58	2.22984E-06
Asr2p	*ASR2*	1.51	7.925E-08
E3 ubiquitin ligase complex SCF subunit	*SKP1*	1.51	1.62635E-05
Coatomer subunit delta	*RET2*	1.49	4.98621E-07
Ribosomal protein P1B	*RPP1B*	1.47	9.63755E-07
1-phosphatidylinositol-3-phosphate 5-kinase	*FAB1*	1.47	2.84155E-07
Non-histone chromosomal protein 6	*NHP6*	1.44	4.4867E-08
Hsp30p	*HSP30*	1.40	2.70703E-05
Histone H3-like centromeric protein CSE4	*CSE4*	1.29	6.90335E-08
Non-specific serine/threonine protein kinase	*SNF1*	1.28	2.23391E-05
Eukaryotic translation initiation factor 5A	*ANB1*	1.27	1.64279E-05
Adenylosuccinate lyase	*ADE13*	1.22	7.23007E-07
Histone H2A.Z	*HTZ1*	1.16	7.40773E-06
Aminomethyltransferase	*GCV1*	1.15	1.74722E-05
Ribosomal protein P2B	*RPP2B*	1.12	7.1798E-07
Glycine cleavage system H protein	*GCV3*	1.10	1.99403E-07
V-type proton ATPase subunit a	*VPH1*	1.05	8.81781E-06
Proteasome endopeptidase complex	*PRE3*	1.01	6.97507E-06
Histone H2B.1	*HTB1*	0.99	8.95516E-07
Transcriptional regulator HMO1	*HMO1*	0.99	3.5035E-06
Ribosomal 40S subunit protein S15	*RPS15*	0.97	4.21377E-05
Mitochondrial intermembrane space import and assembly protein 40	*MIA40*	0.97	1.15022E-05
Homocitrate synthase	*LYS21*	0.97	0.007104253
Heat shock protein SSA2	*SSA2*	0.96	2.03949E-07
Acetyl-coenzyme A synthetase	*ACS1*	0.95	0.012978934
Translation initiation factor eIF5	*TIF5*	0.93	0.029095105
Single-stranded DNA-binding protein	*RIM1*	0.92	2.30606E-07
H/ACA ribonucleoprotein complex subunit CBF5	*CBF5*	0.92	3.18021E-05
Eukaryotic translation initiation factor 3 subunit B	*PRT1*	0.91	1.29315E-05
Protein URE2	*URE2*	0.87	1.6667E-05
Formylglycinamide ribonucleotide amidotransferase	*ADE6*	0.85	9.28313E-05
Slk19p	*SLK19*	0.84	2.96101E-06
SAM complex subunit	*SAM50*	0.84	0.006068136
ATP-dependent DNA helicase II subunit 2	*YKU80*	0.82	0.000547408
40S ribosomal protein S27	*RPS27A*	0.81	0.016012806
RNA polymerase II degradation factor 1	*DEF1*	0.77	3.52533E-06
Transcription factor of morphogenesis MCM1	*MCM1*	0.76	1.27252E-05
Proteasome regulatory particle lid subunit	*RPN5*	0.75	0.038865914
Hsp90 cochaperone	*STI1*	0.75	3.45556E-05
Adenine phosphoribosyltransferase	*APT1*	0.74	0.006011399
Ras GTPase-activating-like protein IQG1	*IQG1*	0.74	0.00184567
Cys-Gly metallodipeptidase DUG1	*DUG1*	0.72	2.76378E-06
FK506-binding protein 3	*FPR3*	0.72	0.001494096
Nucleosome assembly protein 1	*NAP1*	0.71	5.43861E-05
RuvB-like helicase 1	*RVB1*	0.69	0.000441487
Pyrroline-5-carboxylate reductase	*PRO3*	0.69	0.02525234
SUMO family protein	*SMT3*	0.68	4.52665E-05
Galactokinase	*GAL1*	0.66	0.007244711
Ribosomal protein L37	*RPL37B*	0.66	2.54103E-05
V-type proton ATPase subunit C	*VMA5*	0.64	0.001168801
cAMP-dependent protein kinase catalytic subunit	*TPK2*	0.63	2.65332E-05
2-methoxy-6-polyprenyl-1,4-benzoquinol methylase, mitochondrial	*COQ5*	0.62	0.204793578
60S ribosomal protein L13	*RPL13*	0.62	0.001505826
FACT complex subunit POB3	*POB3*	0.62	0.000331606
Aminopeptidase 2	*APE2*	0.62	7.42869E-05
Cyb2p	*CYB2*	0.61	0.008525198
Type I sorting receptor	*PEP1*	0.61	9.73125E-05

**Table 3:** A list of interesting proteins influenced by β-citronellol treatment.

Protein name	Log_2_ fold change	Description/function	References
*Cellular response to oxidative stress/stress response*			
Superoxide dismutase [Cu-Zn]	−1.28	Cytosolic copper- and zinc-containing superoxide dismutase. Destroys radicals by converting into less damaging hydrogen peroxide.	Hwang et al., 1999
Gst2p	−1.37	Glutathione S-transferase. Cellular response to oxidative stress.	So-Hyoung et al., 2014
Stress protein DDR48	2.15	A cell surface protein that senses and responds to changes in the host environment. It is required for stress response and confers partial antifungal drug resistance. Contributes to the DNA damage response.	Cleary et al., 2012
*Cell wall and cell adhesion molecules*			
Cell wall protein RTB1	−16.69	GPI-anchored cell wall protein required for virulence, mating efficiency, and biofilm formation. Normal disseminated infection, but not intestinal colonization.	Braun et al., 2000; Bennett et al., 2003; White et al., 2007; Ene and Bennett, 2009
Agglutinin-like protein 2	−15.77	Cell surface adhesion protein that promotes yeast-to-host tissue adherence as well as yeast aggregation. Play a critical role in the pathogenesis of *Candida albicans* infections.	Aoki et al., 2012; Fanning et al., 2012; Monroy-Pérez et al., 2012
1,3-beta-glucanosyltransferase PGA4	−0.89	Involved in the elongation of 1,3-beta-glucan chains in the cell wall by internally splits a 1,3-beta-glucan molecule and transfers the newly generated reducing end (the donor) to the non-reducing end of another 1,3-beta-glucan molecule (the acceptor). Involved in cell wall biosynthesis and morphogenesis.	Plaine et al., 2008; Ene et al., 2012
*ATP synthesis*			
ATP synthase subunit gamma	−0.72	Mitochondrial proton-transporting ATP synthase complex, catalytic sector *F*(1). Play an important role in proton motive force-driven ATP synthesis.	Gaudet et al., 2011
ATP synthase subunit d, mitochondrial	−1.23	Mitochondrial membrane ATP synthase (F_1_F_0_ ATP synthase or Complex V) produces ATP from ADP in the presence of a proton gradient across the membrane which is generated by electron transport complexes of the respiratory chain.	Gaudet et al., 2011
Cytochrome *c* oxidase subunit 1	−0.79	Component of cytochrome *c* oxidase, the final enzyme in the mitochondrial electron transport chain responsible for oxidative phosphorylation.	Hartley et al., 2019
Cytochrome *b*	−0.91	Component of the ubiquinol-cytochrome *c* reductase complex in the mitochondrial respiratory chain (cytochrome *b*-c1 complex). Involved in the transfer of electrons from ubiquinol to cytochrome *c*.	Brivet-Chevillotte and di Rago, 1989; Beattie et al., 1994

### Biofilm Formation Assay

**Figure 4:**
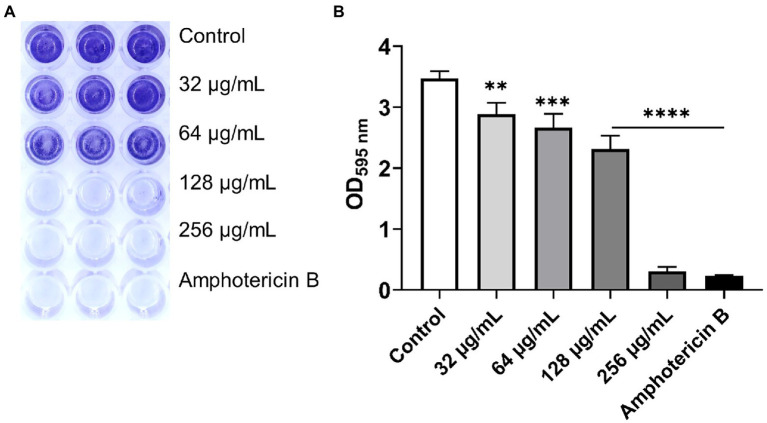
Biofilm formation assay of *Candida albicans*. **(A)** Crystal violet staining of *Candida albicans* biofilm treated with various concentrations of β-citronellol and amphotericin B (2 μg/ml) for 24 h. **(B)** Absorbance at 595 nm of destaining solution from all treatment conditions. Data are represented as mean ± SD. ^**^p ≤ 0.01, ^***^p ≤ 0.001, and ^****^p ≤ 0.0001 compared to the control.

The aim of this experiment was to determine the effect of β-citronellol on yeast biofilm formation. Biofilm formation of *C. albicans* was altered after β-citronellol treatment, as shown in Figure 4. The lowest concentration (32 μg/ml) of β-citronellol significantly suppressed the formation of yeast biofilm compared to the control. The inhibitory effect was higher when concentration increased, and the result at the highest concentration (256 μg/ml) was comparable to amphotericin B treatment.

### Production of ROS

**Figure 5:**
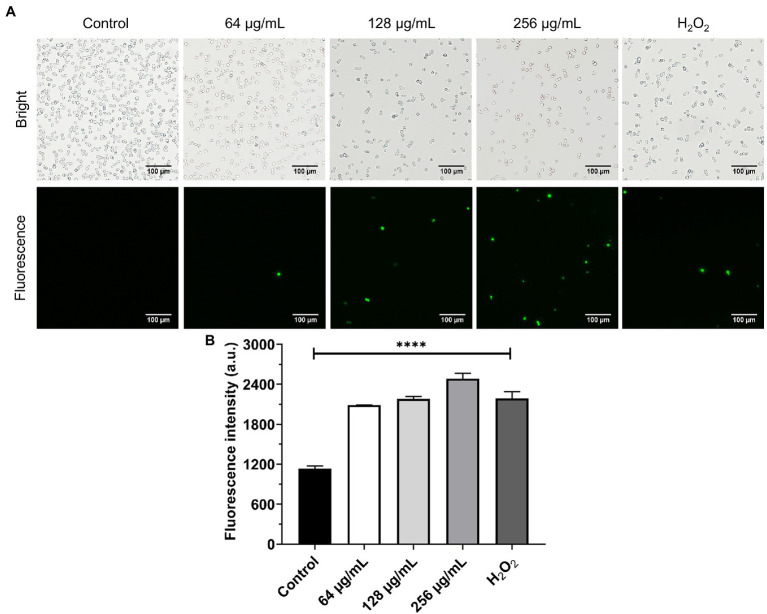
Effect of β-citronellol on ROS production. Approximately 1 × 10^6^ cells/mL were treated with β-citronellol and H_2_O_2_ (0.2 mM) for 4 h. **(A)** Morphology of yeast under bright field and fluorescence microscope. **(B)** Fluorescence intensity of the H_2_DCFDA probe at excitation/emission wavelength 485/535 nm. Data are represented as mean ± SD. ^****^p ≤ 0.0001 compared to the control.

In this experiment, the effect of β-citronellol on induced ROS production was investigated. Production of ROS as a key factor in apoptosis was evaluated using an H_2_DCFDA probe. Results are shown in Figure 5. *C. albicans* treated with β-citronellol for 4 h showed an increase of ROS production by upregulation of green fluorescence intensity compared to the control group. ROS production increased in a dose-dependent manner.

### Cell Membrane Potential

**Figure 6:**
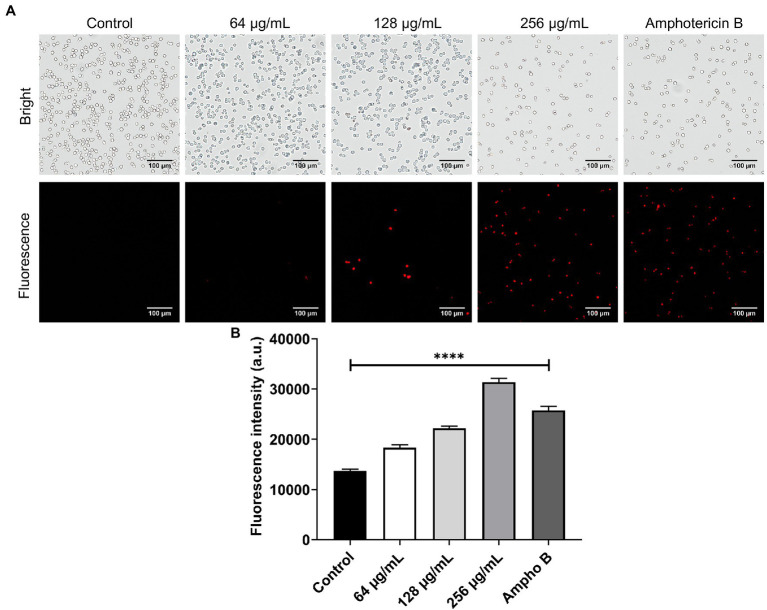
Alteration of the cell membrane of *Candida albicans* in β-citronellol treatment. Yeast cells (1 × 10^6^ cells/ml) were treated with β-citronellol and amphotericin B (2 μg/ml) for 4 h. **(A)** Morphology of yeast cells under bright field and fluorescence microscope. **(B)** Red fluorescence intensity of the DiBAC_4_(3) probe at excitation/emission wavelength 492/518 nm. Data are represented as mean ± SD. ^****^p ≤ 0.0001 compared to the control.

The effect of β-citronellol on yeast membrane potential was determined using a DiBAC_4_(3) probe. Fluorescence dye enters cells through depolarized membrane binding to intracellular proteins or membrane, resulting in a fluorescence signal. As shown in Figure 6, red fluorescence in β-citronellol treated groups significantly increased in a dose-dependent manner compared to the control, indicating the alteration effect of β-citronellol on the cell membrane.

### Apoptosis Analysis

**Figure 7:**
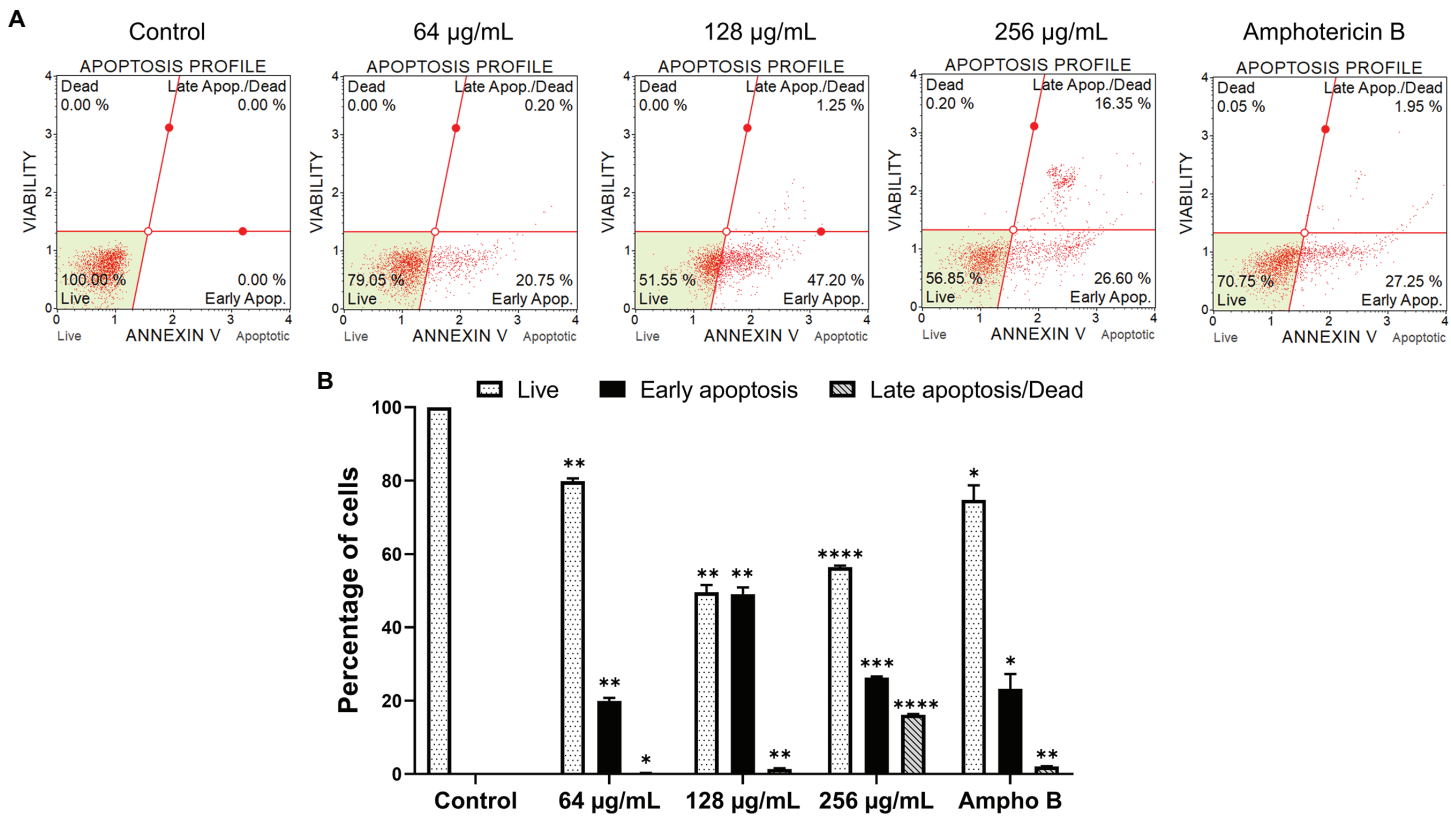
Effect of β-citronellol on *Candida albicans* apoptosis. Cells were incubated with β-citronellol (64–1,256 μg/ml) and amphotericin B (2 μg/ml) for 6 h. **(A)** Chromatogram of the apoptosis profile of *Candida albicans* in different conditions. **(B)** Rate of positive cells in each phase including live, early apoptosis and late apoptosis/dead. Data are represented as mean ± SD. ^*^p ≤ 0.05, ^**^p ≤ 0.01, ^***^p ≤ 0.001, and ^****^p ≤ 0.0001 compared to the control.

The goal of this study was to determine the apoptosis profile of yeast cells after being exposed to β-citronellol. The apoptosis-inducing effect of β-citronellol is shown in Figure 7. After incubating the cells at concentrations of 64, 128, and 256 μg/ml for 6 h, the rate of early apoptosis increased to 20.75, 47.20, and 26.60%, respectively compared to the control. For late apoptosis/dead positive cells, the rate demonstrated a dose-dependent manner ranging from 0.20, 1.25% and maximum at 16.35% in β-citronellol treatment at 64, 128, and 256 μg/ml, respectively. The rate of dead or necrotic cells was positive for 0.20 and 0.05% in 256 μg/ml of β-citronellol and amphotericin B treated conditions, respectively. Results showed that 6 h β-citronellol treatment caused *C. albicans* programmed cell death, primarily activating the early to late apoptosis phases.

### Morphological Change by SEM Observation

**Figure 8:**
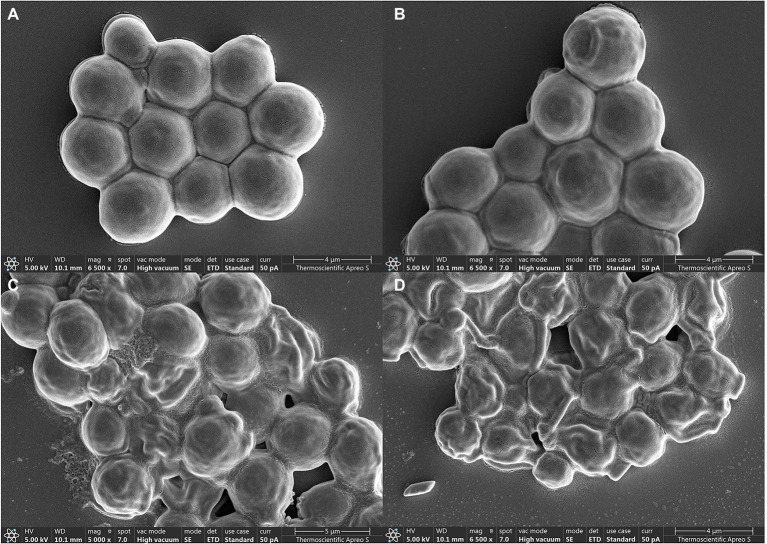
Electron microscopy of *Candida albicans*. **(A)** Typical structure of yeast cells under normal condition. Structural change was observed after incubation with 64 μg/ml **(B)**, 128 μg/ml **(C)**, and 256 μg/ml **(D)** of β-citronellol.

Morphological and structural changes of *C. albicans* under β-citronellol treatment were observed by SEM, with results shown in Figure 8. The yeast cells had spherical shapes and smooth surfaces (A). After 24 h of β-citronellol treatment, the cells exhibited wrinkled surfaces with irregular shapes (B–D), this result could indicate damage to the cell wall and/or cell membrane. The wrinkles increased as the concentration of β-citronellol increased.

## Discussion

*Candida* spp. are common opportunistic pathogens affecting immunocompromised patients worldwide. Various species in this genus are responsible for infection. *C. albicans* is the most frequently isolated, and regarded as the primary etiologic agent in invasive fungal diseases affecting humans, with an unacceptable mortality rate (Firacative, 2020). Furthermore, emerging drug resistance strains are a source of concern that demand researchers to devise novel treatment strategies. Previous reports identified promising anticandidal agents derived from plants, such as β-citronellol (Sharma et al., 2020a,b). However, data on the molecular mechanism of action in terms of global protein expression remains scarce. This study aimed to fill the research gap by identifying proteins that play an important anticandidal role under β-citronellol treatment, using a proteomic approach.

In this study, the MIC and MFC values of β-citronellol against *C. albicans* ATCC 90028 were found to be 128 and 256 μg/ml, respectively. The R-(+)-β-citronellol isomer had a similar MFC value to *C. albicans* ATCC 76645 but a two-fold lower MIC value, according to previous studies. Another isomer, S-(−)-β-citronellol, had MIC and MFC values four and two times lower, respectively (Silva et al., 2020). These characteristics, as well as the varied strains of *Candida* utilized, may contribute to the different MIC and MFC values due to the limitation of not being able to identify the β-citronellol isomer. Proteomic study indicated three distinct categories of target proteins that could be involved in the mechanism of action of β-citronellol in killing *C. albicans*. The first group is cell wall-associated proteins, such as Als2p, Rbt1p, and Pga4p. The cell wall of *C. albicans* consists of two layers. The outer layer is a protein coat consisting mainly of glycosylphosphatidylinositol (GPI) proteins, while the inner layer is formed of polysaccharides consisting of β-1, 6-glucan, β-1, 3-glucan, and chitin (Klis et al., 2009). Several reports have described GPI proteins, including agglutinin-like sequence (ALS), and Rbt1p. These proteins are required for attachment to host epithelial cells, biofilm formation, mating efficiency, and virulence (Staab Janet et al., 1999; Ene and Bennett, 2009; Monniot et al., 2013; Hoyer and Cota, 2016). In addition, the treatment suppressed the expression of a key enzyme involved in the elongation of 1,3-beta-glucan chains, Pga4p (Plaine et al., 2008; Ene et al., 2012). Interestingly, previous study demonstrated a possible mechanism for two isomers of β-citronellol targeting the *C. albicans* cell membrane but not the cell wall (Silva et al., 2020). Additionally, two previously mentioned proteins, Als2p and Pga4p, have been annotated as being membrane- and cell wall-localized (Cabezón et al., 2009; UniProtKB, 2022a,b). As a result, we hypothesize that β-citronellol suppression of both Als2p and Pga4p proteins may lead to a decrease in the cell membrane stability and eventually cell death. As a result, downregulation of these proteins may contribute to reduce biofilm formation (Figure 4), alteration of the membrane potential (Figure 6), and the wrinkled surface structure seen under SEM (Figure 8). These findings suggest that β-citronellol can alter the cell membrane stability, inhibit cell wall biosynthesis, and impede biofilm formation. However, our research did not go into adequate detail on the distinct isomers and their biofilm inhibitory effect. In addition, further research is needed to completely understand the many isomers of β-citronellol as well as the proteins involved in the biofilm development process.

The cellular oxidative stress response mechanism was disrupted by exposure to β-citronellol. In comparison to the control, the treatment condition increased intracellular ROS accumulation by raising the green fluorescence intensity (Figure 5). The result is consistent with a decrease in the expression of Sod1p, a crucial antioxidant enzyme (Table 2). In *C. albicans*, six genes encode for SODs; *SOD1* or cytosolic copper- and zinc-containing superoxide dismutase, two manganese-dependent superoxide dismutases as *SOD2* and *SOD3* localized in mitochondria and cytosol, respectively, and three copper-zinc-containing superoxide dismutases as *SOD4*, *SOD5*, and *SOD6* that are GPI-anchored cell wall-associated enzymes (Martchenko et al., 2004; Sun et al., 2017). The data suggested that β-citronellol suppressing Sod1p may result in insufficient antioxidant activity to deal with ROS accumulation. Furthermore, ROS production induces cell damage and is implicated in the apoptosis process (Perrone et al., 2008), which correlates with increased cell apoptosis in a dose-dependent manner as seen in Figure 7, as well as activation of heat shock proteins (Ssa2p, Hsp30p, and Sti1p). One of the other stress response proteins, Ddr48p, increased in the treatment state. Ddr48p is a crucial protein for detecting environmental changes and partial antifungal treatment resistance in the host (Cleary et al., 2012). In contrast to Gst2p, there was a decrease in β-citronellol treatment in *C. albicans*. Gst2p is necessary for filamentous growth under nitrogen deficiency, according to a prior study (So-Hyoung et al., 2014). These findings suggested that treating *C. albicans* with β-citronellol could reduce stress adaptation and enhance vulnerability to stress-induced settings, such as nutrition deprivation. Moreover, endoplasmic reticulum (ER) stress was modulated by treatment with β-citronellol. Ca^2+^ depletion leads to ER stress and results in the accumulation of misfolded proteins in the ER, which activates signaling pathways such as the unfolded protein response (UPR) and stimulates Ca^2+^ influx across the plasma membrane. The influx can replenish the Ca^2+^ level in organelles *via* a variety of Ca^2+^ channels, including calcineurin and calmodulin (Cmd1p), and is necessary for long-term survival (Bonilla et al., 2002; Liu et al., 2015). Cmd1p expression was increased in this study, implying that when exposed to β-citronellol, the Ca^2+^ level may decrease, and yeast cells attempt to activate the Ca^2+^ compensation mechanism. Due to the absence of validation experiments in this study, additional research is necessary to confirm the effect of β-citronellol on Ca^2+^ levels in yeast cells modulated by Cmd1p activity.

The other interesting target proteins are the proteins involved in the electron transport chain and ATP synthesis, such as F_1_F_0_-ATP synthase. In fungi, ATP synthesis is mainly produced through oxidative phosphorylation with the key enzyme F_1_F_0_-ATP synthase (Li et al., 2021). Proteins involved in the electron transport chain and ATP synthesis, such as F_1_F_0_-ATP synthase, are also potential targets. ATP generation in fungi is mostly accomplished through oxidative phosphorylation, which is catalyzed by the enzyme F_1_F_0_-ATP synthase (Li et al., 2021). The complex consists of two main domains: a globular F_1_ catalytic domain (the α_3_β_3_ domain), and a membrane-bound F_0_ proton-translocating domain (the ab_2_c_8-12_ domain). The F_1_F_0_ domains are linked with the stalk containing the γ, ε, and δ subunits (Duvezin-Caubet et al., 2003; Song et al., 2018). These complexes produce ATP from ADP *via* coupled proton transport in mitochondria and the δ subunits required for *C. albicans* pathogenicity (Boyer, 1997; Li et al., 2021). This study showed that β-citronellol treatment can suppress the expression of ATP synthase subunit gamma (Atp3p) and ATP synthase subunit d, mitochondrial (Atp7p) compared to control. Furthermore, in the presence of β-citronellol, the proteins Cox1p and Cobp, which are involved in the mitochondrial respiratory chain, were found to be repressed. These data imply that β-citronellol may have a direct effect on ATP synthesis, especially at the electron transport chain step, and may cause cell death. Increased ROS levels, on the other hand, can decrease ATP/ADP carrier activity (Perrone et al., 2008). As a result, further investigation into the interaction of β-citronellol and F_1_F_0_-ATP synthase in *C. albicans* is needed.

Treatment with β-citronellol produces multiple types of cell death, including apoptosis, as shown in Figure 7. The expression of the AP-1-like transcription factor CAP1 (Cap1p), one of the well-studied proteins linked with apoptosis in this investigation, was shown in the unexpected trend. Cap1p is a transcription factor implicated in a number of pathways in *C. albicans*, including multidrug resistance (Alarco and Raymond, 1999), oxidative stress response (Zhang et al., 2000), and apoptosis (Dai et al., 2013). Overexpression of Cap1p decreases apoptosis in *C. albicans* triggered by Baicalein, according to a prior study (Dai et al., 2009). Cap1p also plays a role in apoptosis *via* controlling glutathione reductase gene expression and glutathione levels (Dai et al., 2013). In this study, we identified an elevation trend for Cap1p and apoptosis in the treatment group. Because Cap1p is implicated in the oxidative stress response, yeast cells may increase Cap1p expression to maintain redox equilibrium. Cap1p expression was shown to be higher in *C. albicans* treated with caspofungin in another investigation (Kelly et al., 2009). It is worth noting that caspofunin is an antifungal echinocandin that disrupts cell wall production by targeting β-glucan synthase, and that β-citronellol likewise might affects the yeast cell wall. It is possible that there is a link here. More research is needed, however, to establish the interaction of β-citronellol with yeast cell walls, as well as to fully comprehend the link between Cap1p and cell wall production.

## Conclusion

Our findings show that β-citronellol has a potent anti-*Candida* impact, regulating several aspects of yeast biological activity. Three primary categories of target molecules were emphasized in this discovery as cell wall and cell adhesion, cellular oxidative stress response, and ATP synthesis. Numerous other proteins revealed by the proteomic approach may also be implicated in the action mechanism of this compound, and further studies are needed to fully understand the influence of β-citronellol on yeast molecular signaling networks.

## Data Availability Statement

The datasets presented in this study can be found in online repositories. The names of the repository/repositories and accession number(s) can be found at: http://www.ebi.ac.uk/pride/archive/, PXD030379.

## Author Contributions

KU, CN, RP, and WB: conceptualization. WB, YT, and KD: methodology. SK and YY: software. WB: formal analysis, investigation, and writing—original draft preparation. KU: writing—review and editing, visualization, supervision, and project administration. KU, CN, RP, and PP: funding acquisition. All authors contributed to the article and approved the submitted version.

## Funding

This research was funded by the Thailand Science Research and Innovation (PHD60I0053), and the Thailand Science Research and Innovation, and Naresuan University (FF2566).

## Conflict of Interest

The authors declare that the research was conducted in the absence of any commercial or financial relationships that could be construed as a potential conflict of interest.

## Publisher’s Note

All claims expressed in this article are solely those of the authors and do not necessarily represent those of their affiliated organizations, or those of the publisher, the editors and the reviewers. Any product that may be evaluated in this article, or claim that may be made by its manufacturer, is not guaranteed or endorsed by the publisher.
